# *In-vitro* Morphological Assessment of Apoptosis Induced by Nimbolide; A Limonoid from *Azadirachta Indica (Neem Tree)*

**DOI:** 10.22037/ijpr.2019.2391

**Published:** 2019

**Authors:** Muhammad Kashif, Yawon Hwang, Wun-Jae Kim, Gonhyung Kim

**Affiliations:** a *Veterinary Medical Center, College of Veterinary Medicine, Chungbuk National University, Cheongju, South Korea. *; b *Department of Urology, College of Medicine, Chungbuk National University, Cheongju, South Korea.*

**Keywords:** Nimbolide, Cancer cell lines, Normal cell lines, Apoptosis, Caspase activity

## Abstract

The present study was designed to investigate the *in-vitro* morphological assessment of apoptotic effect caused by nimbolide on the selected cancer cell lines (DU-145, PC-3, A-549) and normal cell lines (NIH3T3, CCD-18Co). The cells were grown in 6 well tissue culture plates after treatment with different concentrations of nimbolide and untreated control cells. Apoptotic and necrotic cells were measured using Hoechst 33342 and propidium iodide dual staining through a fluorescent microscope and also by staining with annexin V and propidium iodide through flow cytometric analysis. The activity of caspase 3, 8, and 9 was measured by caspases colorimetric assay kits. The number of apoptotic and necrotic cells were significantly higher in all selected cancer cell lines treated with nimbolide as compared with untreated control cells, whereas in normal cell lines no significant difference was observed between nimbolide treated and untreated cells. The activity of caspase 3, 8, and 9 was also significantly higher in all cancer cell lines treated with nimbolide as compared with untreated control cells while it did not change significantly in normal cell lines as compared with untreated control. The results of the present study suggested that nimbolide induced apoptosis only in cancer cells without affecting the normal cells and one of the apoptosis inducing mechanism is through the activation of caspases signaling pathways. Therefore, nimbolide may be a novel promising candidate as an anticancer drug in future.

## Introduction

Cancer is a major killer disease in the human beings all over the world. About 14 million new cases emerged and 8.2 million deaths occurred due to cancer on a worldwide scale during 2012. This number is predicted to be increased to 22 million within the next two decades (1). The present modes of cancer treatment including surgery, chemotherapy, and radiotherapy can reduce only 5% cancer-related deaths (2). Moreover, these are expensive, toxic and can modify normal genes functions as compared with traditional therapeutic agents. Due to unavailability of suitable cancer treatment, there is a dire need to develop novel therapeutic agents. Therefore, extensive research is currently being focused on finding better chemotherapeutic agents from natural existing plants which can prevent or suppress carcinogenesis. Currently, more than 60% anticancer drugs are derived in one way or another from natural sources (3). Natural compounds had attracted a lot of attention as cancer therapeutics and cancer prevention agents (4). Previous studies have demonstrated that natural compounds play a major role in cancer treatment and prevention through modulating signaling pathways of apoptosis, anti-inflammatory effects, and antioxidant activity (5, 6). 

Azadirachta indica (locally called neem), also known as “Nature’s drug store”, “Village dispensary”, and “Tree of forty” in Africa, is a fast-growing, evergreen plant, resistant to high temperature and drought, widely distributed throughout the Asian countries and a source of various useful products (7). The leaves, seeds, bark, and branches of this plants are used as traditional medicine and several compounds of medicinal importance have reported (8). Neem phytoconstituents have been shown to possess biological and pharmacological activities including antibacterial, antiviral, antipyretic, antihistaminic, anti-inflammatory, anti-tubercular, spermicide, antiprotozoal, analgesic, antiarrhythmic, antimalarial, spermicidal, diuretic and anti-hormonal (9–11). Neem components can also block and suppress cancer growth through induction of cell cycle arrest, interfering with growth signaling pathways, induce apoptosis, decrease tumor volume through angiogenesis, migration and invasion (12, 13). Each part of neem tree produces diverse phytochemicals which have the presence of various isoprenoids and non-isoprenoids such as flavonoids, coumarins, terpenoids, carbohydrates, proteins, sulphurous compounds, polyphenols, fatty acids and their esters (14). Nimbolide is a tetranortriterpenoid, also known as limonoids with a δ lactone ring and an α, β unsaturated ketone system which is derived from leaves and flowers of neem tree and has been used for a variety of diseases as traditional folk medicine (15, 16). Previous studies have shown that nimbolide exhibits several biological activities of antimicrobiological, antimalarial, antifeedant, and antitumor properties (17-20). 

The toxicity of the compounds to normal cells have always been an issue in its therapeutic use. The neem tree leaves and flowers are widely consumed by people and animal in many parts of the world (21). In a previous study conducted in our lab, it was found that nimbolide only exert a cytotoxic effect on the selected cancer cells but did not show significant toxicity to normal cells *in-vitro* (22). Apoptosis induced by many bioactive components from natural sources is considered a very important tool in the treatment and prevention of cancer (23). Therefore, the present study aims to investigate the presence of apoptosis induced by nimbolide in selected cancer cell lines and normal cell lines along with the mechanisms involved in induction of apoptosis. 

## Experimental

*Nimbolide preparations*

Nimbolide from *Azdirachta indica* of purity ≥98% was purchased from Sigma Aldrich, USA (catalog no. SMB000 586). The stock solution 1 of nimbolide was prepared by dissolving 5 mg of nimbolide in 1 mL of dimethyl sulfoxide (DMSO) making a final concentration of 10.72 mM. The stock solution II was made by dissolving 45 uL from stock solution 1 into 955 uL of Dulbecco’s Modified Medium (DMEM) making a final concentration of 500 uM. The stock solution II was diluted prior to use with medium to obtain the desired concentration of nimbolide. 

*Chemicals*

Trypan blue (catalogue no. T6146), Propidium iodide stain (catalog no. p4864), Hoechst 33342 stain (catalog no. 23491-45-4) were obtained from Sigma, USA. FLICE/Caspase 8 colorimetric assay kits (catalogue no. K 113-100), FLICE/Caspase 3 colorimetric assay kits (K106-100), FLICE/Caspase 9 colorimetric assay kits (catalogue no. K 119-100) were purchased from Bio Vision, USA. Dulbecco’s Phosphate Buffered Saline (D-PBS) (catalogue no. PBS-1A), Fetal Bovine Serum (FBS) (catalogue no. S 001-01), Penicillin-streptomycin (catalogue no. LS202-02) and Trypsin-EDTA (catalogue no. LS015) were purchased from Welgene, South Korea, DMEM (catalogue no. DMEM-HPA), and RPMI-1640 (catalogue no. RPMI-A) medium from Capricorn Scientific, South Korea, MTT dye (Thiazolyl Blue Tetrazolium Bromide, Amresco®) (catalogue no. 0793-59) from Solon Ind. Pkwy, DMSO (catalogue no. 67-68-5) was obtained from Junsei, Japan, FITC annexin-V/Dead cell apoptosis kit (catalogue no. V13242) was purchased from Invitrogen, USA.

*Cell lines and cell culture*

The cancerous cell lines A-549, PC-3, Du-145, and noncancerous NIFH3T3 and CCD-18Co were purchased from Korean cell line bank. A-549 is lung cancer cell line, Du-145, PC-3 are prostate cancer cell lines, NIH3T3 is mouse embryonic fibroblast cell line and CCD-18Co is a colon fibroblast cell line. 

The cancerous cells were grown in RPMI 1640 media and non-cancerous cells were maintained in DMEM using 25 cm^3 ^tissue culture flask. At confluence, the cells were transferred to 75 cm^3 ^tissue culture flask according to manufacturer’s instructions. All the cell lines were supplemented with 10% heat-inactivated FBS and 1% antibiotics and maintained at 37 °C in humidified 5% CO_2 _and 95% air 

incubator.

*Morphological changes in Phase-contrast Microscopy*

To observe the morphological changes, each cancer cell line and normal cell line was grown using 6 well tissue culture plates after treatment with different concentrations of nimbolide (0, 2.5, 5 and 10 uM) and vehicle (DMSO 1 uL/mL) for 24 h and 48 h and then observed under phase contrast microscope (24). 

*Propidium Iodide-Hoechst double staining*

The cells were treated with different concentrations of nimbolide (0, 2.5, 5 and 10 uM) and grown in 6 well tissue culture plate (5 × 10^5^ cells/well) for 24 h and 48 h. After that, the cells were trypsinized by adding 1 mL of trypsin-EDTA (0.25%) and put in a CO_2 _incubator for 5 min. Trypsinization was terminated by adding complete medium and the cells were centrifuged. After washing with PBS, the cells were suspended in Hoechst 33342 solution (10 ug/mL) and incubated for 15 min in dark. Afterwards, the cells were mixed and counterstained by adding propidium iodide solution (50 ug/mL) and placed in a CO_2_ incubator for 15 min. The cells were centrifuged and the suspended cells were placed on a clean glass slide and observed immediately under a fluorescence microscope (S46; Microscopes, Inc. St. Louis, MO, USA) using dual excitations. The cells having blue chromatin with organized structure were considered normal cells, while the cells that had bright blue stained highly condensed or fragmented chromatin were considered as early apoptotic cells. On the other hand, the cells with apoptotic nuclei that had bright pink, highly condensed or fragmented chromatin were considered as late apoptotic cells and yet the cells with normal nuclei that had pink chromatin with organized structure were declared as necrotic/dead cell. All the cells were stained in triplicate. At least 300 cells were counted and quantified for percentage apoptosis and necrosis according to the following formula (25, 26).

$\mathrm{Percentage of Apoptotic Cells}=\frac{\mathrm{Early apoptotic cells}+\mathrm{late apoptotic cells}\times 100}{\mathrm{Total number of cells}}$

$\mathrm{Percentage of Necrotic Cells}=\frac{\mathrm{Dead Cell}\times 100}{\mathrm{Total number of cells}}$

*Flow cytometry analysis*

Apoptosis was also detected by staining with annexin V and propidium iodide by using the FITC annexin-V/Dead cell apoptosis kit (Invitrogen, USA) according to the manufacturer’s instruction. Briefly, the cells were treated with nimbolide (5 uM) and grown in 6 wells tissue culture plate for 24 h. After that, cells were washed with PBS and suspended in 100 uL 1x annexin binding buffer (prepared by adding 1 mL of 5x annexin buffer into 4 mL deionized water), 5 uL of FITC annexin V and 1 uL of the propidium iodide (100 ug/mL) working solution to each 100 uL of cell suspension. 

The cells were incubated for 15 min at room temperature. Subsequently, 400 uL of 1x annexin binding buffer was added, mixed gently and analyzed by flow cytometer equipped with an argon ion laser tuned to 488 nm wavelengths (Becton Dickinson FACS Calibur, Heidelberg, Germany). 

*Caspase 3, Caspase 8 and Caspase 9 Activity Assay*

The activity of caspase 3, 8, and 9 was measured using caspases/FLICE colorimetric assays kits. These assays are based on the spectrophotometric detection of the chromophore p-nitroaniline after cleavage from the labeled substrates DVED- pNA, IETD-pNA and LEHD-pNA for caspases 3, 8, and 9, respectively. Briefly, the cells were treated with nimbolide (5 uM) for 24 h. After that the cells were trypsinized and pelleted (1 x 10^6 ^cells) in all groups. 

The cell pellets were resuspended in a 50 uL chilled cell lysis buffer and incubated for 10 min on ice. Lysed cells were centrifuged at 10,000 × g at 4 °C for 1 min. Lysate proteins in equal amounts from each sample were added to 96 wells tissue culture plate, 50 uL of 2X reaction buffer (containing 10 mM DTT) and 5 uL pNA-conjugated substrates (DEVD-pNA, IETD-pNA and LEHD-pNA; substrates for caspases 3, 8 and 9, respectively) were also added and placed at 37 °C for 2 h in CO_2_ incubator according to manufacturer’s instructions. The amount of pNA released was measured at 405 nm using an ELISA microplate reader (Emax, Molecular Device, Sunnyvale, CA, USA). Each of the caspase activity was expressed by the value of OD 405. 

*Statistical analysis*

All the experiments were performed in triplicates and the data were presented as means ± standard deviation. Statistically significant difference between treated cells and untreated control cells was analyzed through one-way analysis of variance (ANOVA) using IBM SPSS Statistics 24, followed by posthoc analysis. *p* < 0.05 was considered statistically significant. 

## Results

*Antiproliferative effect of Nimbolide*

In the previous study in our lab, we found nimbolide significantly reduced viability of cancer cells as compared with controls in time and dose-dependent manner but did not significantly affect normal cells as compared with the normal cells (22). 

*Morphological observations of selected cell lines using phase contrast microscope*

**Figure 1:**
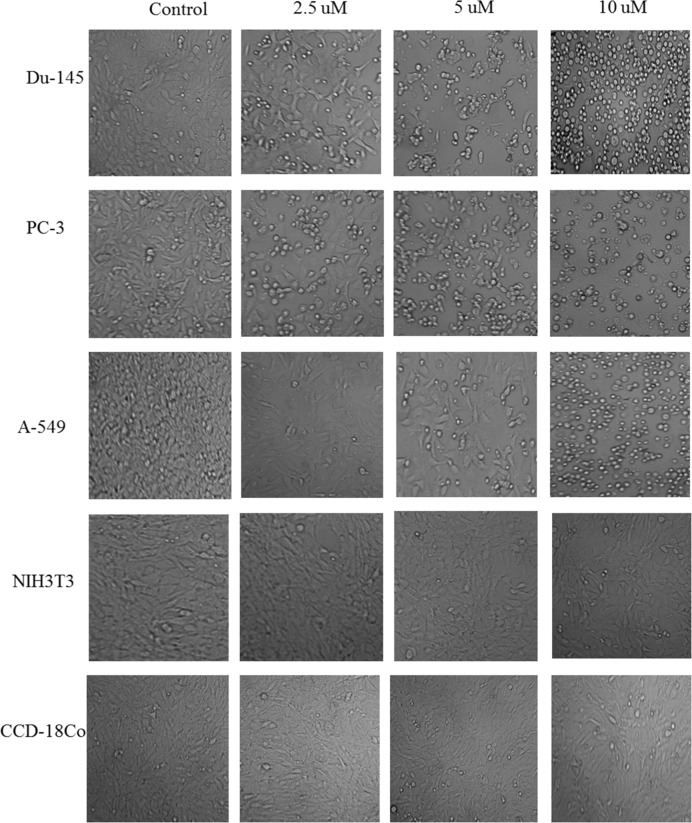
Morphological changes in selected cancer cell lines (Du-145, PC-3, A-549) and normal cell lines (NIH3T3, CCD-18Co). The cells were treated with different concentrations of nimbolide (0, 2.5, 5, and 10 uM) for 48 h and observed under phase contrast microscope (magnification 100x)

After treatment with nimbolide for 24 h and 48 h, morphological changes in all cell lines were observed in comparison to control (untreated) cells under phase contrast microscope. Cancer cells treated with nimbolide showed typical apoptotic features such as decreased number of cells, shrinkages, and membrane blebbing loose contact with adjacent cells, and at high dose even detached from the surface of tissue culture plate and appeared as floating cells in culture medium. Contrary to it, the untreated cells maintained original morphology form and adhere to the tissue culture plates. Normal cells treated with nimbolide and untreated cells did not exhibit typical features of apoptosis under phase contrast microscope. The cells treated with vehicle (DMSO) did not show any effect on the viability and morphology and appeared similar as untreated control cells both in cancer and normal cell lines (Figure 1).

**Figure 2:**
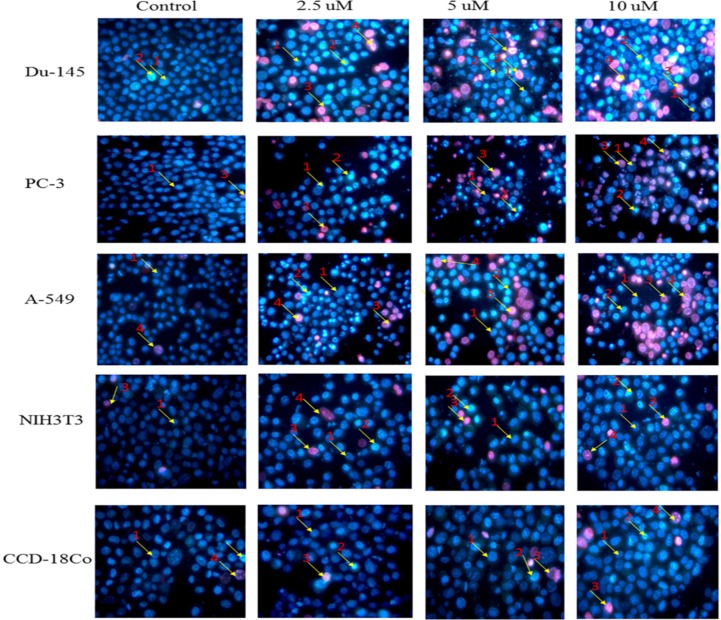
Morphological changes in selected cancer cell lines (Du-145, PC-3, A-549) and normal cell lines (NIH3T3, CCD-18Co) detected with dual staining of Hoechst 33342 and propidium iodide. The cells were treated with different concentrations of nimbolide (0, 2.5, 5, and 10 uM) for 48 h and observed under fluorescent microscope (magnification 400x). Arrows indicate (1) viable cells with normal nuclei, (2) live cells with apoptotic nuclei, (3) dead cells with normal nuclei, and (4) dead cells with apoptotic nuclei

**Figure 3:**
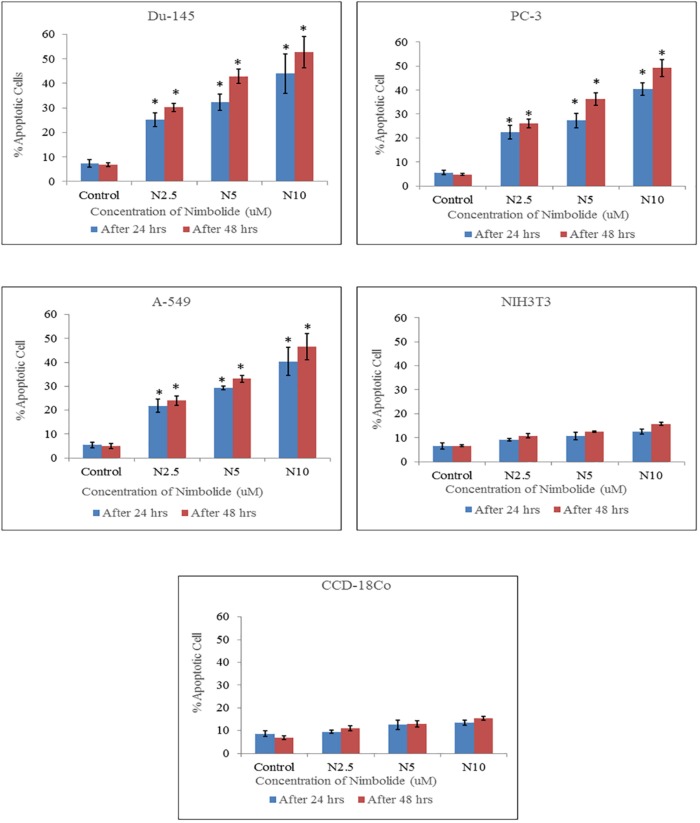
Percentage of apoptotic cells quantified by Hoechst 33342 and propidium iodide dual staining. Both cancer cell lines (Du-145, PC-3, A-549) and normal cell lines (NIH3T3, CCD-18Co) were treated with different concentrations of nimbolide (0, 2.5, 5, and 10 uM) for 24 h and 48 h. The values were represented as means ± standard deviation. *indicates statistically significant difference between treated and control group (*p *< 0.05)

**Figure 4:**
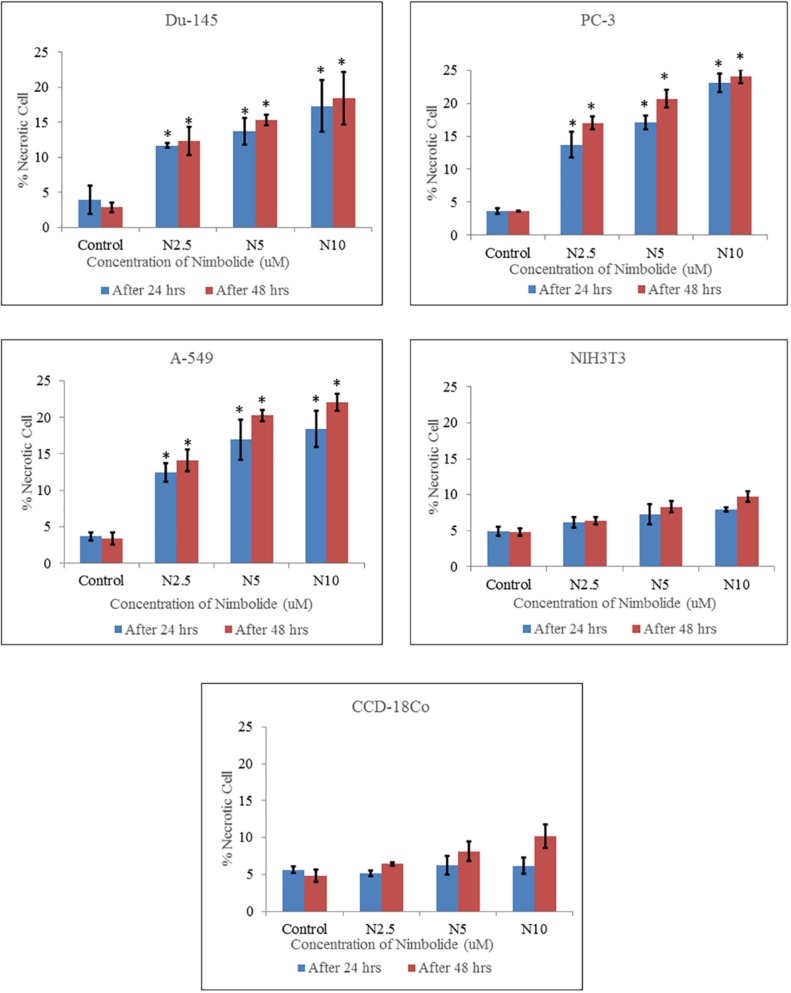
Percentage of necrotic cells quantified by Hoechst 33342 and propidium iodide dual staining. Both cancer cell lines (Du-145, PC-3, A-549) and normal cell lines (NIH3T3, CCD-18Co) were treated with different concentrations of nimbolide (0, 2.5, 5, and 10 uM) for 24 h and 48 h. The values were represented as means ± standard deviation of three independent experiments. *indicates statistically significant difference between treated and control group (*p *< 0.05)

**Figure 5:**
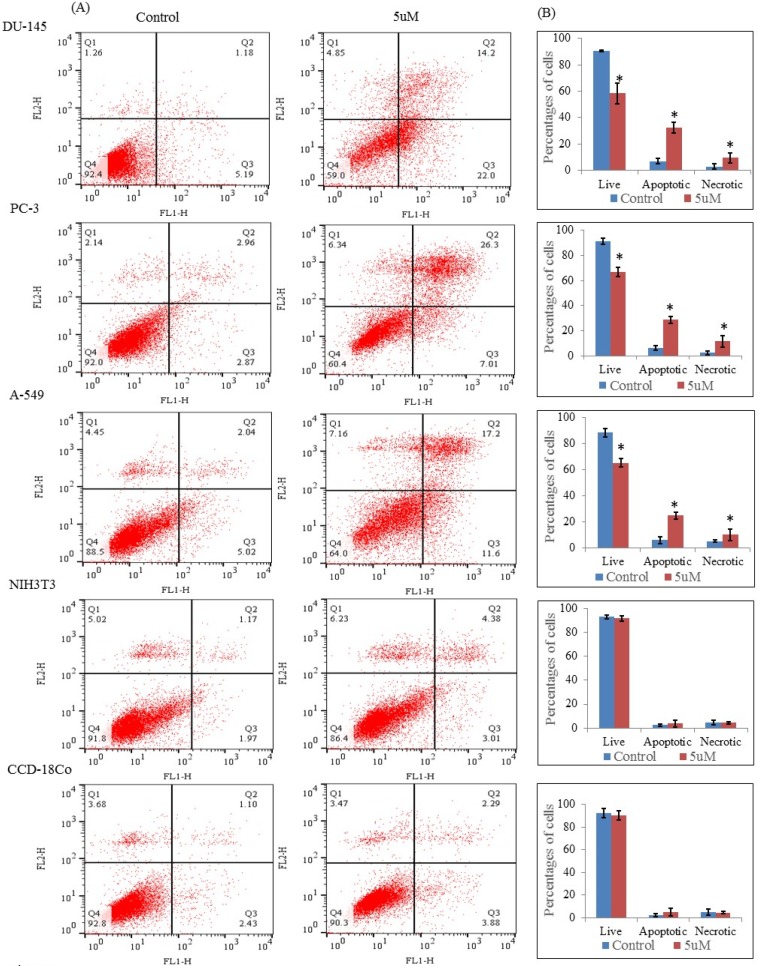
Flow cytometric analysis through Annexin V-FITC and propidium iodide dual staining. The cells were treated with nimbolide (5 uM) for 24 h, then stained with Annexin V-FITC and propidium iodide and measured by flow cytometry. (A) Representative dot plots from one of three independent experiments with percentages of cells in the respective quadrants are indicated Q1: Necrotic cells, Q2: late apoptotic cells, Q3: early apoptotic cells, Q4: live cells. (B) Bar graph represents the average percentages of live, apoptotic (early and late) and necrotic cells ± standard deviation from three independent experiments

*Indicates statistically significant difference between treated and control group (*p *< 0.05).

**Figure 6:**
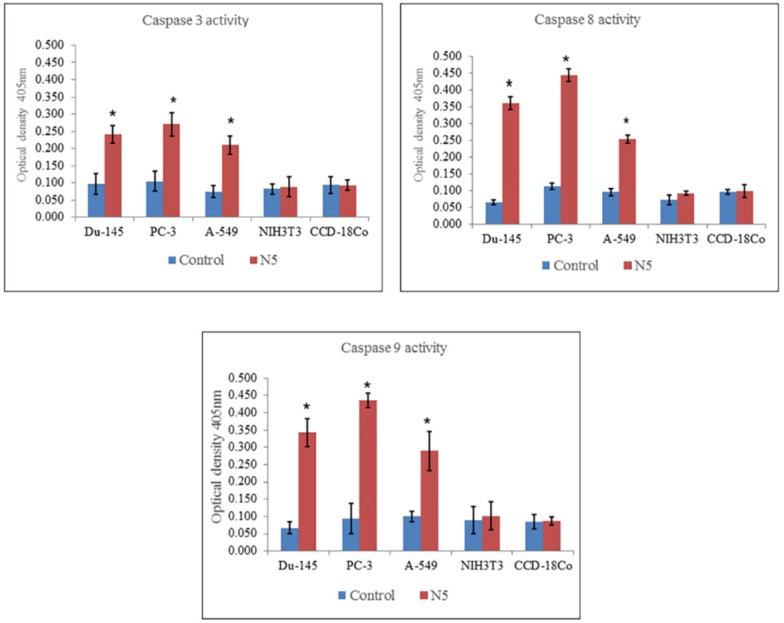
Measurement of caspase 3, 8, and 9 activity of selected cancer cell lines (Du-145, PC-3, A-549) and normal cell lines (NIH3T3, CCD-18Co) through colorimetric assay kits treated with nimbolide (5 uM) for 24 h. Each bar represents the means ± standard deviation of three independent experiments

*Indicates statistically significant difference between treated and control group (*p *< 0.05).

*Morphological observations of selected cell lines using Fluorescent Microscope*

Apoptosis was also assessed and quantified using the dual staining of Hoechst 33342 and propidium iodide with the help of fluorescence microscope. Morphological observation of cancer cells treated with nimbolide for 24 h and 48 h showed significant alterations as compared to untreated control. The cell treated with nimbolide showed chromatin condensation, multiple fragmentations of nuclei, brightly stained with Hoechst 33342 and emitted pink fluorescence (late apoptosis and necrosis) whereas the control (untreated) cells appeared oval shaped, less brightly stained and absence of red fluorescence. The normal cells treated with the same dose of nimbolide did not show significant morphological alteration as compared with untreated control cells under fluorescence microscope (Figure 2). 

The percentages of apoptotic and necrotic cells were significantly higher in nimbolide treated groups as compared with control group in cancer cell lines, whereas, no significant difference was observed between nimbolide treated groups and control group in normal cell lines as depicted in (Figures 3 and 4) respectively. 

*Flow cytometric analysis*

The apoptosis induced by nimbolide was further confirmed by Annexin V and propidium iodide staining assay. The results showed that nimbolide significantly decreased the percentages of live cells (Q4) with increased percentages of the early and late apoptotic cells (Q3 and Q2) and necrotic cells (Q1) in all selected cancer cell lines treated with nimbolide compared with untreated control whereas no significant difference was observed between treated and untreated cells in normal cell lines (Figures 5A and 5B). Thus, it was evident that nimbolide induced apoptosis only in cancer cells without affecting the normal cells.

*Effect of nimbolide on the signal pathway of caspases*

The mechanism of cell death induced by nimbolide was further elaborated by the activity of caspase 3, 8, and 9 measured in both cancer and normal cell lines. The results indicated that nimbolide significantly increased the activity of caspase 3, 8, and 9 in all the selected cancer cell lines as compared with control but did not significantly affect normal cell lines as compared with control (Figure 6). This suggested the involvement of caspases in triggering apoptosis in cancer cells treated with nimbolide.

## Discussion

Previous studies have reported the *in-vitro* antitumor effect of neem tree extracts and nimbolide on different cancer cell lines. Neem leaves extract and its limonoids exerted an antiproliferative effect on the growth of tumor cells *in-vitro* (27, 28). Ethanolic neem leaf extract exerted an inhibitory effect on proliferation of prostate cancer cells *in-vitro* and induced apoptosis (29). Nimbolide reduced the viability of NIE-155 and 143B TK cell lines with the IC_50 _value 4.75 (30). The cytotoxic effect of nimbolide on U937 (leukemic), B16 (melanoma), THP-1 and HL-60 cell lines was observed by Roy *et al*. (2007). Nimbolide exerted an antiproliferative effect on different cancer cell lines (HL- 60, SW-480, HeLa, BeWo cells) through the induction of apoptosis (20, 31). In the present study to the best of our knowledge, we compared for the first time the *in-vitro* morphological apoptosis induced by nimbolide on selected cancer cell lines and the normal cell lines.

Apoptosis (programmed cell death) is an active form of cell death that plays an important role in homeostasis and maintenance of tissue development (32). The tumor cells evade the processes of apoptosis due to defect in their ability to activate the death signaling pathways. Therefore, induction of apoptosis has been described as standard and important target in cancer therapy (33). Apoptosis eliminates malignant or cancer cells without damage to surrounding cells and normal cells (34). Apoptotic cells displayed typical common features such as cell shrinkage, nuclear condensation, membrane blebbing, chromatin cleavage and formation of pyknotic bodies of condensed chromatin (24). In the present study, the similar morphological changes were observed under phase contrast microscope in all the cancer cells when treated with different concentrations of nimbolide for 24 h and 48 h, while none of such changes were observed in the normal cells with the same concentration. This confirms that nimbolide induced apoptosis only in the cancer cells. 

Hoechst 33342 and propidium iodide double staining can be used to observe other apoptotic features such as nuclear fragmentation and chromatin condensation using fluorescence microscopic analysis. Hoechst 33342 is a blue fluorescence dye that stains DNA chromatin while propidium iodide cannot enter into the intact plasma membrane and is only permeable to the dead cell. Therefore, it is possible to differentiate among the live, apoptotic and necrotic cells populations under fluorescence microscope by the staining pattern resulted from the simultaneous use of these dyes (35, 36). In the present study morphological apoptosis was also assessed using dual staining of Hoechst 33342 and propidium iodide through a fluorescence microscope. All of the cancer cells treated with different concentrations of nimbolide clearly showed typical features of apoptosis such as apoptotic bodies, multiple fragmentations, and condensed chromatin that were uniformly brightly fluorescent due to Hoechst 33342. The cells which were in late apoptosis and necrosis emitted pink fluorescence due to propidium iodide. The untreated cancer cells and normal cells treated with a same concentration of nimbolide did not exhibit significant morphological changes under a fluorescence microscope. The apoptotic and necrotic cells were also quantified by careful counting. The percentages of apoptotic and necrotic cells were significantly higher as compared with control in all cancer cell lines treated with nimbolide while a non-significant difference was observed between treated and untreated cells in normal cells lines. Nimbolide induced apoptotic and morphology changes in all cancer cells in a time and dose dependent manner. The exposure of 10 uM nimbolide triggered nearly 50% apoptosis after 48 h in all cancer cell lines. 

Annexin V is a Ca^2+ ^dependent phospholipid-binding protein that has a high affinity for phosphatidylserine. In apoptotic cells, phosphatidylserine translocated from inner to the outer layer of the cell membrane and annexin V can identify the apoptotic cells by binding to phosphatidylserine exposed on the outer surface. Propidium iodide that stained only dead cells by binding tightly to nucleic acid was used to identify the necrotic cells. In the present study, the cells were stained with annexin V and propidium iodide and analyzed by flow cytometery to distinguish live, apoptotic, and necrotic cells. The results also confirmed the increased number of apoptotic and necrotic cells in all selected cancer cell lines treated with nimbolide but no significant effect was determined in normal cell lines. However, the apoptotic cells hallmarked increased as compared with necrotic cells in all the cancer cell lines. This confirmed that the main mechanism of cell death is through apoptosis. The presence of necrotic cells with early apoptotic cells suggest that these cells are resulted from an apoptotic process rather than a necrotic process (37). The appearance of necrotic cells may also be due to the incomplete process of apoptosis (38). Therefore, in the present study, the presence of increased necrotic cells as compared with untreated cells may have resulted after apoptotic process or incomplete apoptosis due to increased or decreased concentration; or this may be connected with the more complex effect of nimbolide.

The magnitude of side effect is the most important factor in chemotherapy (39). The data of current study showed that nimbolide induced morphological changes and apoptosis in all selected cancer cell lines but did not exert a significant effect on the normal cell lines. Our study results are in accordance with previous morphological studies by Lajimia *et al.* (40) which described that leaf extract of *Scrophularia striata* only inhibited the proliferation of cancer cell lines while did not exert a significant effect on normal human fibroblast cell line. Venom derived peptides (ICD-85) also inhibited the proliferation of cancer cell lines while no effect was noted when same concentration was exposed to MRS-5 Normal cells (41).

Apoptosis is generally mediated by caspases cascades, a family of cysteine containing aspartate specific proteases, that lead to activation and cleavage of molecules and cause cell death (42, 43). There are mainly two pathways of apoptosis *i.e.* extrinsic and intrinsic pathways. Each of them requires specific triggering signals to begin energy dependent cascades and activate initiator caspase 8, 9, 10 which in turn activates the executioner caspase 3 (44). Caspase 8 is the major extrinsic pathway protein, once activated, it cleaves and activates other caspases and cause the release of cytochrome C that initiates death receptor mediated apoptosis (45, 46). Caspase 3, which is common in both pathways, is an important member and key executor in the apoptotic signaling cascades. It usually exists in the cytoplasm in the form of an inactive zymogen and becomes activated by many external apoptotic signals. It can induce the activation of key proteases in the cytoplasm, nucleus, and cytoskeleton and induce apoptosis of cells (47). The results of the present study showed that treatment of all selected cancer cells with nimbolide significantly increased the level of caspase 3, 8, and 9 activity as compared with untreated control. It did not significantly increase caspase 3, 8, and 9 activity in normal cells as compared with control. The results of the present study confirmed that nimbolide induce apoptosis in cancer cells through activation of caspases by both extrinsic and intrinsic pathways of apoptosis but it does not induce apoptosis in normal cells.

Although sometimes the results obtained *in-vitro* experiments may not fully or accurately predit the effect on a whole organism but biomedical researchers use different methods including experimental animals, tissue, and cell cultures for clinical studies to treat human diseases and disorders. Cancer cell lines have been widely used for research purposes, proved to be a useful tool in the genetic approach and an excellent model for the study of the biological mechanisms involved in cancer and providing information about the deregulated genes and signaling pathways in this disease (48). Furthermore, the *in-vitro* cell model was used in the origin of the development and testing of anticancer drugs presently used and in the development of new therapies (49, 50). In fact, the use of the appropriate *in-vitro* model in cancer research is crucial for the investigation of cell proliferation, cellular pathways, apoptosis, deregulation, and cancer progression, to define potential molecular markers and for the screening and characterization of cancer therapeutics (51). The results of the research of *in-vitro* models are usually extrapolated to *in-vivo* human tumors as models for drug testing and have been recognized by many biomedical and pharmaceutical companies (52). 

In conclusion, in the light of the findings of the current study, it is suggested that the function of nimbolide to induce apoptosis is selective on cancer cells without affecting the normal cells and one of the apoptosis inducing mechanism of nimbolide is through activation of caspases signaling pathways. Further clinical trials and *in-vivo* studies are needed to develop it as an anticancer drug. Nimbolide may be a new hope as an effective anticancer drug in future.
